# Synchronized Oviposition Triggered by Migratory Flight Intensifies Larval Outbreaks of Beet Webworm

**DOI:** 10.1371/journal.pone.0031562

**Published:** 2012-02-09

**Authors:** Yun Xia Cheng, Li Zhi Luo, Xing Fu Jiang, Thomas W. Sappington

**Affiliations:** 1 State key Laboratory for Biology of Plant Diseases and Insect Pests, Institute of Plant Protection, Chinese Academy of Agricultural Sciences, Beijing, China; 2 United States Department of Agriculture -Agricultural Research Service, Corn Insects & Crop Genetics Research Unit, Genetics Laboratory ISU, Ames, Iowa, United States of America; Australian Wildlife Conservancy, Australia

## Abstract

Identifying the reproductive consequences of insect migration is critical to understanding its ecological and evolutionary significance. However, many empirical studies are seemingly contradictory, making recognition of unifying themes elusive and controversial. The beet webworm, *Loxostege sticticalis* L. is a long-range migratory pest of many crops in the northern temperate zone from 36°N to 55°N, with larval populations often exploding in regions receiving immigrants. In laboratory experiments, we examined (*i*) the reproductive costs of migratory flight by tethered flight, and (*ii*) the reproductive traits contributing to larval outbreaks of immigrant populations. Our results suggest that the beet webworm does not initiate migratory flight until the 2nd or 3rd night after emergence. Preoviposition period, lifetime fecundity, mating capacity, and egg hatch rate for adults that experienced prolonged flight after the 2nd night did not differ significantly from unflown moths, suggesting these traits are irrelevant to the severity of beet webworm outbreaks after migration. However, the period of first oviposition, a novel parameter developed in this paper measuring synchrony of first egg-laying by cohorts of post-migratory females, for moths flown on d 3 and 5 of adulthood was shorter than that of unflown moths, indicating a tightened time-window for onset of oviposition after migration. The resulting synchrony of egg-laying will serve to increase egg and subsequent larval densities. A dense population offers potential selective advantages to the individual larvae comprising it, whereas the effect from the human standpoint is intensification of damage by an outbreak population. The strategy of synchronized oviposition may be common in other migratory insect pests, such as locust and armyworm species, and warrants further study.

## Introduction

Migration is an important life history option for adapting to seasonal and temporal changes in habitat [1]–[3]. A migrant reduces the risk of extinction of its genotype in the natal habitat by spreading its offspring to more suitable habitat patches [2]–[5]. However, migration may impose reproductive costs on the migrant relative to residents [6]–[9]. Development of a flight apparatus in migrants, such as flight muscles or even wings, along with the expense of fuel usage during the energetically demanding process of flight may reduce lifetime fecundity [6], [7], [9] by diverting resources from egg development. Similarly, male migrants may be less competitive for females [10], [11]. In addition, a relatively long preoviposition period (POP) for migrants, a trait that often accompanies migratory behavior [2], [6]–[9], [12], [13], may decrease the total number of days available for reproduction. Although trade-offs between flight and reproduction may be common, this conclusion has been based mostly on observations from wing-dimorphic insects. In wing-monomorphic insects this relationship may be different. For example, in the Glanville fritillary butterfly, *Melitaea cinxia*, lifetime fecundity was higher in the more dispersive females than in the less dispersive individuals [14]. The more dispersive morph may compensate for the energy cost of flight with increased food intake after flight [14], accounting for its higher metabolic rate than that of less mobile individuals [15]. Furthermore, the reproductive consequences after flight may be substantially different from those of unflown migratory-phase adults, because reproduction and its governing physiological processes usually do not occur until after migration [2], [12], [13]. For instance, in the wing-dimorphic cricket *Gryllus texensis*, the mating capacity and ovary weight of the flight-capable morph is generally poorer than that of the short-winged morph, but is comparable when measured after the former has experienced flight [11], [16].

Studies on the reproductive consequences of flight have been mostly focused on lifetime fecundity, ovarian development or length of the POP, and mating capacity. Inferences of cost depend on the species and the reproductive trait investigated. Lifetime fecundity may be decreased, as in the fruit fly, *Drosophila melanogaster* [6], and noctuid moths, *Heliothis virescens*, *Pseudoplusia includens* and *Spodoptera exempta* [17]–[19], or increased, as in the milkweed bug, *Oncopeltus fasciatus*, and migratory grasshopper, *Melanoplus sanguinipes* [20], [21]. Lifetime fecundity of the black bean aphid, *Aphis fabae* [22], and beet armyworm, *Spodoptera exigua* [23] is unchanged by flight, while that of the chloropid fly, *Oscinella frit*, and oriental armyworm, *Mythimna separata* varies with flight age [24], [25]. In some species, flight promotes ovarian development or shortens the length of POP [13], [18], [24]–[26]. In males, flight may enhance mating behavior [16], [27], [28] or have no effect on mating capacity [23], [25]. These findings illustrate why the nature of reproductive consequences of migration have defied generalization and remain controversial. Moreover, reproductive traits affected by migration that, in turn, affect population development are largely unknown and unexplored. Reaching an overarching understanding of the evolutionary and ecological consequences of insect migration will require addressing such issues.

The beet webworm, *Loxostege sticticalis* L. (Lepidoptera: Pyralidae), is a destructive pest of crops and fodder plants grown in many areas of the northern temperate zone from 36°N to 55°N, including North America, Eastern Europe and Asia [29]–[33]. The species causes economically-serious outbreaks over large areas in northern China [31], [33]. Migration by *L. sticticalis* is an adaptation to seasonal and regional variation in environmental elements [31], [34]–[37], and often has major ecological consequences. Simultaneous outbreaks of larval populations may extend over 10 million hectares, and they occur in areas that have received a huge influx of immigrant adults [33], [38]–[40]. Although the known migratory and reproductive behaviors of adult *L. sticticalis* may play important roles in the resulting outbreaks of larval populations, we suspected that the reproductive capacity of *L. sticticalis* moths after migration may be enhanced. This is because captures of 10,000 and 5,000 adults per night by a single blacklight trap (1.5 m high, one per county in both source and immigrant areas of northern China, according to the National Standard of Monitoring and Forecasting of *L. sticticalis*) have been used in China since the 1980s as thresholds for predicting outbreaks in source and immigrant areas, respectively [41], [42]. In other words, it takes only half the number of adults to produce an outbreak after migration than before migration. We also hypothesized that the reproductive consequences to *L. sticticalis* moths mediated by migratory flight could perhaps serve to promote outbreaks among progeny of a cohort of immigrants. If so, we do not know which reproductive trait is improved and how the outbreaks of larval populations are enhanced, because there are no reports on the reproductive consequences for *L. sticticalis* moths after migration.

We therefore studied the interplay between migratory flight and reproduction with an eye to possible mechanisms that could promote larval population outbreaks. We first determined the likely migratory flight age based on flight and reproductive data from adults of different ages that experienced tethered-flight of various periods. We then evaluated the effects of migratory flight on general reproductive traits and a novel parameter that serves as a measure of population-level synchrony of egg-laying, the period of first oviposition (PFO). Finally, we discussed the role that each post-migration reproductive trait may play in enhancing outbreaks of *L. sticticalis* larvae.

## Results

### Flight Performance

**Figure 1:**
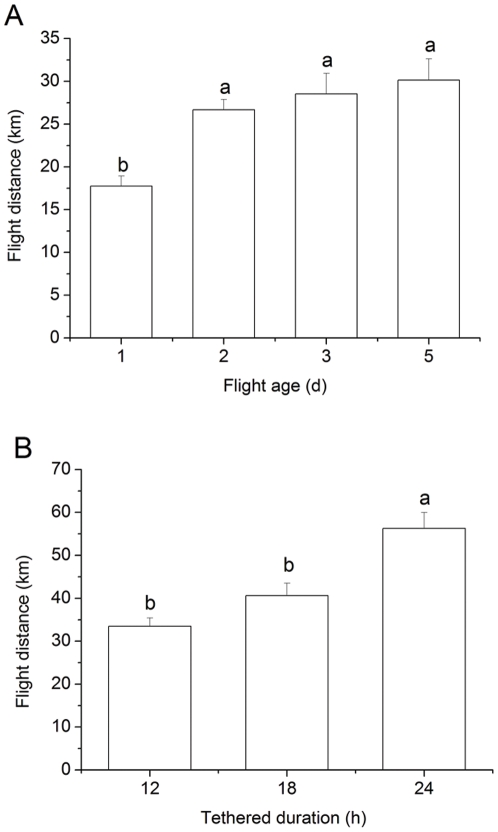
Flight distance of adult *L. sticticalis* during a 12-h tethered-flight test at different ages (A), and during different flight test durations at 3 d of age (B). Data are presented as mean ± SEM. Bars sharing the same letter are not significantly different at 5% level by Tukey's HSD test. Sample sizes for each treatment in panel A are 34, 38, 28 and 34, and in panel B are 38, 36 and 34, from left to right, respectively.

The flight distance (*F*_3, 130_ = 7.36, *P*<0.0001) of *L. sticticalis*, as determined by tethered-flight technique during a 12-h test period, varied greatly with moth age (Fig. 1A). Distance flown by 1-d-old adults was significantly less than that of 2-d-, 3-d- and 5-d-old adults (*P* = 0.008; *P* = 0.003; *P*<0.0001), but did not significantly differ among the latter three age groups (*P*>0.50). Flight distance of 3-d-old adults also proportionally and significantly increased as the flight test period extended from 12 h to 24 h (*F*_2, 105_ = 16.17, *P*<0.0001) (Fig. 1B). Moths of the 24-h flight test flew significantly farther than those in the 12-h and 18-h tests (*P*≤0.001), but the flight distances reached by the12-h and 18-h flight groups were not significantly different (*P* = 0.07).

### Effects of flight on general reproductive traits

**Figure 2:**
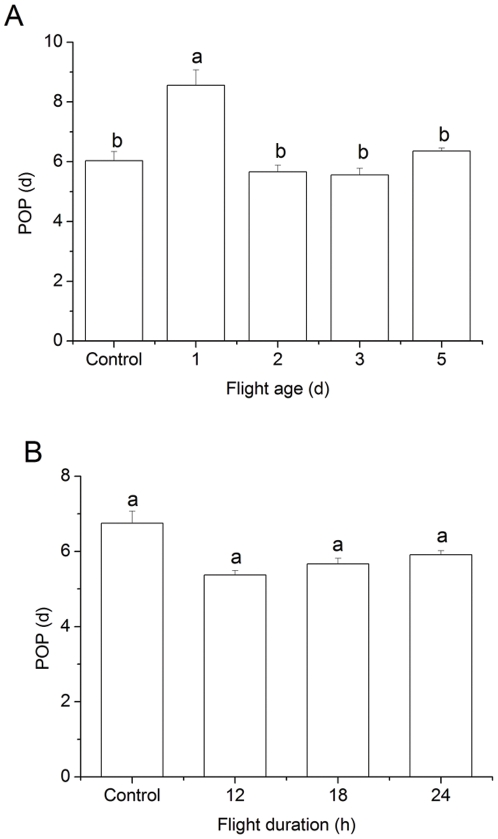
Preoviposition period (POP) of adult *L. sticticalis* that experienced a 12-h tethered-flight test at different ages (A), and different flight test durations at 3 d of age (B). Data are presented as mean ± SEM. Bars sharing the same letter are not significantly different at 5% level by Tukey's HSD test. Sample sizes for each treatment in panel A are 26, 25, 27, 25 and 30, and in panel B are 32, 27, 28 and 24, from left to right, respectively.

POP was significantly affected by age at which the adult was flown (*F*_4, 132_ = 11.00, *P*<0.0001), ranging from 5.5 to 8.6 d (Fig. 2A). POP in adults flown at d 1 was 8.6 d, which was ∼2.2 d greater than that of the other 4 treatments (*P*<0.0001). The POP of adults flown at d 2, 3 and 5, did not differ significantly from each other or from the control moths (*P*>0.05). Mean POPs of the 3-d-old adults in the experiment testing extended flight periods ranged from 5.4 to 6.7 d (Fig. 2B), and did not differ significantly from each other (*F*_3, 107_ = 0.37, *P* = 0.78).

Mean lifetime fecundity of moths flown at different ages ranged from 318 (d5) to 363 (d2), but none of the differences were significant (*F*_4, 100_ = 1.59, *P* = 0.18) (Table 1). Similarly, there were no significant differences among any treatment groups in the age study for mating frequency (*F*_4, 100_ = 1.46, *P* = 0.22), percentage of moths that mated (control vs. flown, *χ* ^2^ = 0.16, *df* = 1, *P* = 0.69), and egg hatch rate (*F*_4, 72_ = 2.25, *P* = 0.07) (Table 1). In the experiment testing for effects of extended flight period on 3-d-old moths, there were no significant differences in lifetime fecundity (*F*_3, 89_ = 0.96, *P* = 0.413), mating frequency (*F*_3, 89_ = 0.58, *P* = 0.63), percentage of moths that mated (control vs. flown, *χ* ^2^ = 0.27, *df* = 1, *P* = 0.60), and egg hatch rate (*F*_3, 83_ = 0.40, *P* = 0.76) (Table 2).

**Table 1:** Reproductive performance of *L. sticticalis* after experiencing a 12-h flight test at different ages of adult life.

Flight age (d)	Life time fecundity	Mating frequency	Mating percentage (%)	Egg hatch rate
Control	351.63±13.01a (19)	1.26±0.10a (19)	74.07	0.55±0.05a (15)
1	319.26±18.71a (19)	1.11±0.07a (19)	75.00	0.62±0.05a (14)
2	363.26±18.25a (23)	1.35±0.13a (23)	75.00	0.72±0.04a (16)
3	324.14±14.64a (21)	1.48±0.11a (21)	85.71	0.71±0.04a (14)
5	318.43±17.02a (23)	1.30±0.10a (23)	75.76	0.66±0.06a (18)

Data are presented as mean ± SEM. Number in parentheses is the corresponding sample size. In each column, data sharing the same letter are not significantly different at the 5% level by Tukey's HSD test. The mating percentage between all of the flown groups pooled (since there is no significant difference in mating percentage among flown groups) and the unflown group is not significantly different, as determined by a Chi-square test (*χ* ^2^ = 0.16, *df* = 1, *P* = 0.69).

**Table 2:** Reproductive performance of *L. sticticalis* after experiencing different flight test durations on d 3 of adult life.

Flight duration (h)	Life time fecundity	Mating frequency	Mating percentage (%)	Egg hatch rate
Control	314.55±25.34a (22)	1.32±0.12a (22)	72.97	0.77±0.02a (22)
12	321.64±18.98a (22)	1.45±0.17a (22)	78.57	0.79±0.03a (21)
18	337.36±16.36a (25)	1.56±0.13a (25)	71.43	0.78±0.03a (24)
24	303.33±16.86a (24)	1.38±0.13a (24)	87.50	0.81±0.03a (20)

Data are presented as mean ± SEM. Number in parentheses is the corresponding sample size. Data in a column sharing the same letter are not significantly different at 5% level, as determined by Tukey's HSD test. The mating percentage between all of the flown groups pooled (since there is no significant difference in mating percentage among flown groups) and unflown treatments is not significantly different, as determined by Chi-square test (*χ* ^2^ = 0.27, *df* = 1, *P* = 0.60).

### Effects of flight on period of first oviposition (PFO)

**Figure 3:**
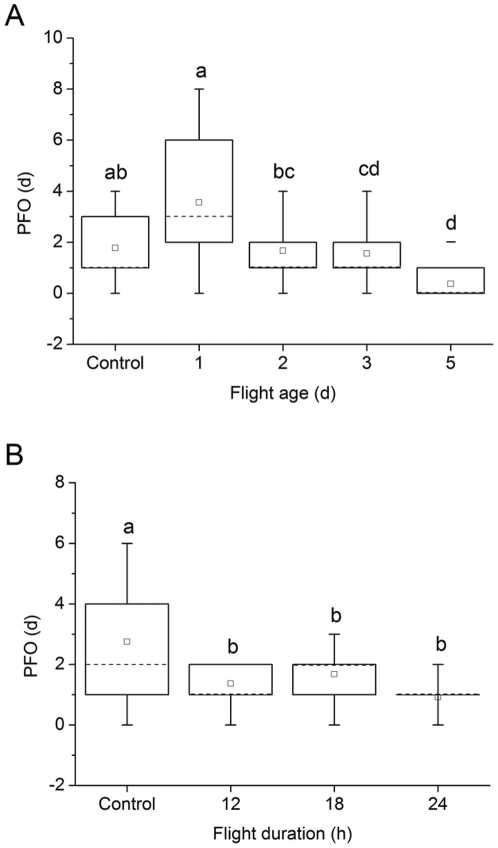
The period of first oviposition (PFO) of adult *L. sticticalis* that experienced a 12-h tethered-flight test at different ages (A) and different flight test durations at 3 d of age (B). Data are presented (from top to bottom in each of the box-and-whiskers plots) as the maximum (-), upper quartile (—), mean (□), median (---), lower quartile (—), and the minimum (-). Means with the same letters in each panel are not significantly different at 5% level by Tukey's HSD test. Sample sizes for each treatment in panel A are 26, 25, 27, 25 and 30, and in panel B are 32, 27, 28 and 24, from left to right, respectively.

PFO of adult *L. sticticalis* was significantly affected by age at time of flight (*F*_4, 128_ = 16.91, *P*<0.0001) (Fig. 3A). Moths flown at d1 of adult life needed, on average, 3.6 d to initiate oviposition once the first moth in that group began ovipositing, which was significantly greater than those flown at ages 2, 3 and 5 (*P*<0.0001), but it was not significantly prolonged over that of the control (*P* = 0.06). Mean PFOs of moths flown at d 3 and d 5 were significantly shorter than that of unflown control moths (*P*<0.05). Mean PFOs between the paired treatments of d 2 and control, d 2 and d 3, d 3 and d 5 were not significantly different (*P*>0.05), but that of the moths flown at d 5 was significantly shorter than that of the moths flown at d 2 (*P* = 0.01). Thus, the timing of initial oviposition was more synchronous in moths flown at 3–5 d of age than among those flown at 1 d of age or among those not flown at all.

Among 3-d-old moths, mean PFO was significantly affected by flight test duration (*F*_3, 107_ = 13.60, *P*<0.0001) (Fig. 3B), which was consistent with results of the previous experiment. Mean PFOs of the moths that experienced the 12-, 18-, and 24-h flight test were significantly less than that of the unflown control moths (*P*≤0.002), but did not differ significantly from each other among these 3 flown groups (*P*≥0.08). In other words, initiation of oviposition was more synchronous among moths in the flown treatments than in the unflown controls.

## Discussion

### Flight potential and migratory flight period of *L. sticticalis*

Adult *L. sticticalis* demonstrated great flight capacity in the tethered flight tests of the two experiments (Fig. 1). Tethered flight distance on each day tested was comparable to that of *S. exigua* [23], a noctuid species with one of the greatest documented expanses traversed in migratory flight [43]. Flight capacity of *L. sticticalis* moths was relatively weak on the first day of adult life, then markedly increased on d 2 where it remained high through at least d 5 (Fig. 1A). This finding is consistent with previous results obtained by the same tethered-flight technique [34]. Although 2-d-old adults exhibited similar flight capacity as 3- and 5-d-old moths in the current study (Fig. 1A), their propensity to engage in flight was lower than older moths in a study employing a free-flight recording system [34]. In that study, it was from d 3 on that the *L. sticticalis* moths showed a propensity to fly readily and seemed primed for a long duration flight [34].

The reproductive consequences of *L. sticticalis* moths flown at different ages also support our conclusion that migratory flight does not occur on the first day of adult life. The significant increase in POP of 1-d-old *L. sticticalis* adults experiencing flight compared to unflown controls and adults flown at ages of 2–5 d (Fig. 2A) may indicate a reproductive cost [6], [13], although their lifetime fecundity was not significantly less than that in other treatments (Table 1). Additionally, adult *L. sticticalis* flown at d 1 of the adult life had a greater PFO than the unflown controls and those in the flight treatments (Fig. 3A). This suggests that overnight flight at this age disturbs synchronized onset of oviposition, which will not promote development of an outbreak population (see below). Finally, ovaries of *L. sticticalis* trapped en route and in the immigrant areas are mostly at a stage of development [35], [44] coinciding with that of 3- to 5-d-old adults in this study.

We conclude that adult *L. sticticalis* may spend one or two days after emergence in their natal habitat garnering supplemental nutrition to boost energy reserves, and to allow full development of the flight system before initiating migratory flight. This differs from the case of gregarious-form *S. exempta*, which initiates migration on the night of adult emergence or at dusk the following night [45]. Females can be sexually receptive as early as the second night after emergence [46].

### Response of general reproductive traits to flight

We found no evidence that migratory flight of *L. sticticalis* on d 2–5 of adult life has any effect on POP. This differs from examples where flight promotes ovarian development or shortens the POP, as are the cases in locust and grasshopper [21], [26], a chloropid fly [24], and oriental armyworm [25]. Unchanged POP after an initial flight may allow further migration of *L. sticticalis* moths. Swarms of immigrants usually disappear shortly after descending into areas where humidity and temperature are not suitable for reproduction [38], [42].

Lifetime fecundity of *L. sticticalis* flown on d 2 to d 5 of adult life was not affected (Table 1), a finding that is not uncommon in other species [13], [20]–[25], but which differs from cases where flight decreases lifetime fecundity [6], [17]–[19]. Likewise, flight distance of 3-d-old *L. sticticalis* during extended 18-h and 24-h flight test periods increased proportionally but did not result in fewer eggs (Table 2), suggesting mechanisms for quickly restoring energy for egg production. Maintenance of the egg production potential of *L. sticticalis* moths after flight may be achieved by energy replenishment through feeding, or through resorption of flight muscle, as demonstrated in other insects [17], [19], [20], [47], [48]. Indeed, the number of eggs produced is greatly increased in a few species when the adults can feed after flight [17], [20]–[22].

Our results showed that the mating capacity of both genders of *L. sticticalis* moths was not significantly affected by flight at any age tested (Table 1) or in 3-d-old adults at any test duration up to 24 h (Table 2). Similar results were observed in *M. separata* and *S. exigua*, in which mating capacity of adults flown at d-1 to d-5 of adult life was not different than unflown controls [23], [25]. In contrast, mating ability of male crickets after flight is improved [16], [27], [28]. Egg-hatch rates of beet webworm were not affected by the different flight treatments in either experiment (Table 1, 2), suggesting that neither mating capacity nor fertilization are intrinsically different among flown and unflown moths.

### PFO and its role in outbreaks of *L. sticticalis* after migration

In this study, we employed a new parameter, PFO, to describe the time window of onset of oviposition after flight (Fig. 3). Our results demonstrated that the PFO of *L. sticticalis* adults varies with flight age and test duration. That is, adult *L. sticticalis* flown at d 1 of adult life subsequently initiated oviposition over a longer time window than those flown on d 2–5, suggesting again that it is not suitable to start migratory flight on the first night after emergence. In addition, the PFOs of moths flown at d 3 and 5 were significantly less than that of the unflown controls. Both experiments show a similar trend (Fig. 3), suggesting that the onset of oviposition after migration by moths more than 2 d old is more synchronous than in moths that have not migrated. Thus, PFO seems to be a good parameter for measuring synchronization of oviposition in *L. sticticalis*, although it needs to be verified with field data.

Synchronization of oviposition can be caused by synchronized maturation of gregarious-form immatures, as in the case of migratory locusts [49]. However, to our knowledge, enhanced synchronization of oviposition resulting from a decrease in PFO of adults after migration has not been investigated heretofore in any other migrant insect species. This mechanism could potentially play a critical role in causing or enhancing outbreaks in many migrant insect pests.

It has been a puzzle that only half as many adult *L. sticticalis* in an immigrant area are needed to cause an outbreak as are needed in the source areas [41], [42]. There are three main contributors to the spatial concentration of *L. sticticalis* which precedes a probable larval population outbreak. First, mass take-off, orientation, vertical layering and descent behavior of a cohort of *L. sticticalis* moths [35], [37] helps ensure the adults land together, as in the case of *S. exempta* [45]. Mass deposition of migrating *S. exempta* adults concentrated by weather systems can lead to dense immigrant populations in East Africa [46]. Second, *L. sticticalis* immigrants remain only in areas where the temperature is *ca* 21°C with adequate moisture to reproduce [33], [39], [40], serving to concentrate oviposition in a defined location. Third, adult and larval host preferences for oviposition and feeding, respectively, concentrate eggs and larvae on a few host plant species, such as lambsquarters [50], a dominant weed species in northern China.

However, in addition to these factors promoting spatial concentration, our results indicate that the decrease in PFO triggered by migratory flight of *L. sticticalis* adults increases the outbreak potential of the immigrant population even further by concentrating the temporal window of oviposition. This is because the decreased PFO of moths after flight results in a more rapid increase of larval population density temporally through increased synchronization of egg hatching. PFO may play a role in increasing the potential for outbreaks in other migrant insect species as well, especially where the induction of migratory behavior is similar to that of *L. sticticalis*, such as in migratory locusts and armyworms.

Enhanced larval density is important in the development of outbreak populations of *L. sticticalis*. Presence in the field of velvet-black gregarious phase larvae of *L. sticticalis*, triggered by crowded conditions, is characteristic of outbreak populations [33], [39], [40]. Gregarious phase larvae eat more across a broadened host plant range, develop faster, and are less likely to suffer attacks of natural enemies compared to isolated larvae [30], [32], [51]. A new emigrant population of moths builds up because the flight capacity of adults derived from crowded larvae is enhanced [52]. Together, these factors may maintain continuity in the outbreak cycle of *L. sticticalis*.

## Materials and Methods

### Insects

The colony of *L. sticticalis* used for the experiments had been reared for 5 generations in the laboratory. Larvae were reared at a density of 10 per 650-ml glass jar and fed daily with fresh leaves of lambsquarters (*Chenopodium album* L.). When larvae matured, sterilized soil containing ca 10% water was added to the cages to a depth of 8–10 cm, which served as substrate for cocoon formation. When adults emerged, pair of male and female were transferred into a transparent 245-ml (5×12.5 cm) plastic cage and provided with 10% glucose solution (w/v) *ad libitum*, which was changed daily until the moths were tested. Adults, eggs, larvae and pupae were maintained at a constant temperature of 22±1°C, 75%±5% RH, and photoperiod of L16: D8.

### Tethered-flight technique and treatments

Two interconnected experiments were designed to examine reproductive parameters after flight. The first experiment focused on the effects of flight at different days after emergence on reproductive parameters of the flown moths. This experiment consisted of 5 treatments, which included unflown moths (controls), and those flown at d 1, 2, 3 or 5 of adult life. The flight test period for all age groups was 12 h, corresponding to the normal overnight migratory activity of *L. sticticalis* in the field [35], [37]. The second experiment was designed to test for variation in reproductive parameters of adults flown for more than 12 h, since migratory flight activity of *L. sticticalis* moths occasionally extends past dawn and into daytime in the field [38], [42]. Three-d-old adults were used because it has been proposed as the age at which the migratory journey is initiated [34]. This experiment consisted of 4 treatments, which included the unflown moths, and those tested for 12 h, 18 h, and 24 h, respectively.

Flight tests were conducted using a 32-channel flight mill system. Each moth was tethered to a flight mill following the techniques used in previous studies [34], [52]. Ambient temperature and humidity during each flight test were maintained at 22±1°C and 70% RH, conditions previously determined to be optimal for *L. sticticalis* flight [34], [52]. Total flight distance was used as the flight capacity of adults in each treatment. In all treatments, flight distance was pooled across genders as there is no significant difference in the flight capacity of males and females [34], [52].

### Reproductive parameters

The tether was carefully removed from the moth after completion of the flight test. The moth was paired and transferred into the original plastic cage of 245-ml, and maintained in the rearing chamber together with the control moths. Daily, egg output of moths was recorded, and supplemental food was changed until the adults were dead. At death, the female was dissected and the number of spermatophores in the bursa was determined. This revealed whether a pair was mated and the number of matings per pair, and allowed calculation of the mating percentage and other reproductive parameters.

The POP, PFO, lifetime fecundity, mating frequency, mating percentage, and egg hatch rate were used to evaluate changes in reproduction in response to different flight treatments. These parameters, except PFO and egg hatch rate, were determined following the methods employed in previous studies [31], [53]. PFO describes the duration of the time window (in days) over which first oviposition occurred among individuals of a treatment group relative to the earliest case of oviposition by any moth within that group. For example, a mean PFO of 2 for a treatment group indicates that, on average, females oviposited for the first time 2 d after the first instance of oviposition in that group. The lower the mean PFO, the more synchronous was the onset of oviposition in a treatment group. Egg hatch rate was calculated as the number of newly hatched larvae divided by the number of eggs observed for the adult pair. Data obtained from unmated pairs were excluded from the data pool, except for mating percentage.

### Data analysis

All numeric values are presented as means ± SEM. Egg hatch rate data were arcsine transformed before testing for a normal distribution by the Shapiro-Wilk test. Differences between treatments were evaluated by one-way analysis of variance. Significant differences among multiple means were determined by Tukey's HSD test. Differences in mating percentage between the treatments were compared by Chi-squared tests. All statistical analyses were performed using SPSS version 16.0 software (SPSS, 2007).
